# Psychometric properties of the Chinese version of the Athletic Identity Measurement Scale Plus: A confirmatory study on Chinese adolescents

**DOI:** 10.1371/journal.pone.0345181

**Published:** 2026-04-13

**Authors:** Shaoshen Wang, Ying Shuai, Garry Kuan, Najib Majdi Yaacob, Yee Cheng Kueh

**Affiliations:** 1 School of Sports Management, Shandong Sport University, Shandong Province JN, China; 2 Biostatistics & Research Methodology Unit, School of Medical Sciences, Universiti Sains Malaysia, Kubang Kerian, Kelantan, Malaysia; 3 Exercise and Sports Science Programme, School of Health Sciences, Universiti Sains Malaysia, Kubang Kerian, Kelantan, Malaysia; Southwest University, CHINA

## Abstract

**Background:**

Athletic identity plays a crucial role in athletes’ psychological well-being and performance. Although the Athletic Identity Measurement Scale Plus (AIMSP) effectively assesses this construct in Western contexts, its application in diverse cultural settings remains challenging. This study aimed to validate the Chinese version of AIMSP (AIMSP-C) among young basketball players in mainland China.

**Methods:**

A cross-sectional validation study was conducted with 604 adolescent basketball players (301 males, 303 females; mean age = 15.53 years, SD = 1.42) from secondary schools across Shandong Province, China. Participants completed the 22-item AIMSP-C, which assesses five dimensions of athletic identity using an 11-point Likert scale. The scale underwent rigorous forward-backward translation following standard cross-cultural adaptation procedures. Confirmatory factor analysis (CFA) was performed to examine the structural validity, alongside comprehensive reliability and validity assessments.

**Results:**

CFA supported a five-factor structure (Social Identity, Exclusivity, Self-Identity, Negative Affectivity, and Positive Affectivity) after minor modifications, demonstrating acceptable fit indices (CFI = 0.959, TLI = 0.952, SRMR = 0.083, RMSEA = 0.091 [90% CI: 0.086, 0.096]). The AIMSP-C exhibited strong internal consistency (composite reliability: 0.974–0.989), convergent validity (AVE > 0.50), discriminant validity, and test-retest reliability (ICC: 0.936–0.989). These psychometric properties surpassed those of the original AIMSP.

**Conclusion:**

The AIMSP-C demonstrates robust psychometric properties for assessing athletic identity among Chinese youth athletes. This validated instrument provides researchers and practitioners with a reliable tool for understanding athletic identity in the Chinese sporting context, facilitating evidence-based approaches to athlete development and psychological support.

## Introduction

Athletic identity, grounded in identity theory [1], represents a specific role identity within individuals’ hierarchical identity structure rather than an objective construct. This theoretical framework posits that athletic identity varies in salience and commitment across individuals and contexts [2]. Athletic identity represents a fundamental psychological construct that profoundly influences athletes’ development, performance, and psychological well-being [3,4]. Over the past three decades, this construct has evolved from a simple characterization of athletic role identification to a sophisticated multidimensional framework that shapes athletes’ psychological processes, behavioral patterns, and career trajectories [5,6]. The significance of athletic identity becomes particularly pronounced during adolescence, a critical developmental period characterized by heightened identity exploration and consolidation [7,8]. According to Erikson#39;s psychosocial development theory, adolescents face the central task of resolving identity versus role confusion, during which they actively construct and negotiate multiple role identities, including their emerging athletic identity. For adolescent athletes, this process is particularly complex, as they must simultaneously navigate the general developmental demands of identity formation while establishing and maintaining their athletic role within competitive sporting environments [3,9].

The validation of athletic identity measurement instruments among adolescent populations is essential for several methodological and theoretical reasons. First, adolescent athletes may interpret and respond to identity-related items differently from adult athletes due to their developmental stage; their identity orientations tend to be more fluid and exploratory compared to the more crystallized identities of established adult athletes [8]. Second, the item content of the AIMSP—which includes references to social recognition, emotional responses to sporting experiences, and the prioritization of sport within one#39;s broader identity—aligns well with the salient developmental tasks of adolescence, during which peer validation, emotional regulation, and role experimentation are central concerns [10]. Third, given the increasing emphasis on early talent identification and systematic athlete development programs worldwide, a validated instrument for measuring athletic identity in adolescent populations addresses a critical gap in the existing measurement literature and enables evidence-based psychological support during this formative period [9].

Athletic identity encompasses five distinct but interrelated dimensions: social identity, exclusivity, self-identity, negative affectivity, and positive affectivity. Each dimension contributes uniquely to understanding how athletes develop, maintain, and express their athletic identity across different contexts and developmental stages.

Central to Cieslak II (5) conceptualization of the AIMSP is the recognition that athletic identity is not merely an internal self-perception but a dynamic construct shaped by the continuous interaction between individuals’ own contributions and reinforcement from the social and sporting context. Individuals’ own contributions encompass personal investment in sport, self-referential cognitions about one#39;s athletic role, and emotional responses to sporting experiences. These internal processes are simultaneously reinforced and modulated by the external social and sporting context, including recognition from coaches, peers, and family members, institutional support structures, and the broader cultural value placed on athletic achievement. This interactional perspective is reflected in the five-dimensional structure of the AIMSP, which can be conceptually organized along an internal–external continuum. Self-Identity, Negative Affectivity, and Positive Affectivity represent primarily internal, self-referential dimensions that capture how athletes perceive themselves and emotionally respond to their sporting experiences. In contrast, Social Identity and Exclusivity are more strongly influenced by external factors, reflecting how the social and sporting environment shapes the prominence and boundaries of one#39;s athletic role. Importantly, these internal and external dimensions are not independent but mutually reinforcing: external recognition (Social Identity) strengthens internal self-perception (Self-Identity), while personal emotional engagement (Positive and Negative Affectivity) influences the degree to which athletes allow sport to define their broader identity (Exclusivity).

### Social identity

Social identity, as a primarily externally oriented dimension in Cieslak II (5) framework, reflects how individuals perceive themselves as athletes through the lens of social recognition and their integration within the sporting community [11]. This dimension encompasses both personal identification with the athletic role and recognition from others [12]. Recent research has revealed that social identity in sports extends beyond mere role recognition, influencing team cohesion, leadership dynamics, and performance outcomes [13]. Athletes with strong social identity demonstrate enhanced commitment to training and competition [4], while also showing greater resilience in facing competitive challenges [14]. In team sports like basketball, social identity plays a particularly crucial role as athletes must integrate individual and collective identities while pursuing both personal and team objectives [15].

### Exclusivity

The exclusivity dimension represents the degree to which an individual#39;s self-worth is predominantly determined by their athletic performance [16]. This dimension has emerged as a critical factor in understanding both the benefits and potential risks of strong athletic identification. While high levels of exclusivity often correlate with enhanced motivation and dedication to athletic excellence [4,17], they may also create vulnerabilities during career transitions or injury recovery periods [18]. Recent longitudinal studies have demonstrated that athletes with high exclusivity scores show greater dedication to training but may face significant challenges in developing alternative identities or preparing for post-athletic careers [19,20]. The relationship between exclusivity and psychological well-being appears to be curvilinear, with moderate levels associated with optimal outcomes while extreme levels may predict adjustment difficulties [21].

### Self-Identity

Self-identity, representing a core internal dimension in Cieslak II (5) framework, captures the strength of athletic self-reference in an individual#39;s overall self-concept [22] and constitutes the cognitive structure through which athletes process information and experiences from a self-referential perspective [23]. Contemporary research has expanded our understanding of athletic self-identity#39;s role in psychological functioning, demonstrating its influence on goal-setting behaviors, motivation patterns, and resilience development [24]. Athletes with well-developed self-identity often exhibit higher levels of sports commitment and achievement motivation [25,26]. However, the development of self-identity must be balanced with other aspects of personal growth to prevent overidentification with the athletic role [27]. Longitudinal research has revealed that the stability of athletic self-identity varies across developmental stages, with adolescence representing a particularly critical period for its formation and consolidation [10].

### Negative affectivity

Negative affectivity in athletic identity represents emotional responses to adverse sporting experiences, including performance setbacks, injuries, or career transitions [3]. Recent research has significantly expanded our understanding of this dimension#39;s role in athlete well-being and performance. Studies indicate that athletes with high negative affectivity may experience more significant psychological distress during challenging periods, particularly following injuries or during competitive slumps [28]. Longitudinal investigations have revealed that elevated negative affectivity can predict increased vulnerability to depression and anxiety, especially during major career transitions or following serious injuries [29,30]. Moreover, contemporary research has identified important mediating factors that influence the relationship between negative affectivity and psychological outcomes, including social support systems, coping strategies, and resilience mechanisms [31].

### Positive affectivity

Positive affectivity reflects the enjoyment, satisfaction, and positive emotional experiences derived from athletic participation [5]. This dimension has emerged as a critical factor in understanding sustained athletic engagement and performance excellence. Recent studies have demonstrated that positive affectivity contributes significantly to athlete motivation, engagement, and performance outcomes through various psychological mechanisms [32]. Athletes with high positive affectivity typically demonstrate greater resilience in facing challenges and maintain stronger commitment to their athletic pursuits [33]. Research has also revealed important relationships between positive affectivity and flow states, optimal performance experiences, and enhanced recovery from setbacks [34]. Furthermore, longitudinal studies have shown that positive affectivity plays a crucial role in buffering against burnout and maintaining long-term athletic engagement [35].

### Cross-cultural validation of athletic identity measurement

The universality of the athletic identity construct has been progressively supported through cross-cultural validation studies employing measurement scales across diverse cultural and linguistic contexts. The original Athletic Identity Measurement Scale (AIMS; Brewer, Van Raalte [3]) was initially developed and validated among Western populations and has since served as the foundation for numerous cross-cultural adaptations. Martin, Eklund [22] provided early evidence for the scale#39;s applicability beyond its original context by validating it among athletes with disabilities. Subsequently, the scale has been adapted and validated in a range of non-Western settings: Sohrabi and Shajie [36] validated the AIMS-Plus among Iranian physical education students, supporting a three-factor structure; Li [37] confirmed the original factor structure in a Hong Kong sample; and Kamran and Rafique [38] supported a five-factor structure among Pakistani athletes. In mainland China, Zhang and Shao [39] validated the original AIMS, providing initial evidence for the construct#39;s relevance in the Chinese sporting context, though this version assessed only the cognitive dimensions without affective components.

Collectively, these cross-cultural studies demonstrate that the core dimensions of athletic identity are recognizable across cultural boundaries, supporting the construct#39;s theoretical universality. However, they also reveal culturally specific variations in factor structures, inter-dimensional relationships, and psychometric properties, underscoring the necessity of culturally adapted measurement tools. The present study extends this body of cross-cultural evidence by validating the more comprehensive AIMSP—which incorporates both cognitive and affective dimensions—among adolescent athletes in mainland China, a population and cultural context that remain underrepresented in the athletic identity measurement literature.

### Research objectives

While Zhang and Shao [39] validated the AIMS in mainland China, the AIMSP#39;s five-dimensional structure, including affective components absent in AIMS, requires separate validation. The AIMSP provides a more comprehensive theoretical framework by incorporating both cognitive and emotional aspects of athletic identity [5]. The validation of AIMSP-C is particularly crucial given China#39;s unique sports system that integrates athletic training with academic education. This “sports-education integration” system creates distinctive challenges for young athletes developing their athletic identity, especially in team sports like basketball, where players must balance multiple roles.

The present study aims to validate the Chinese version of AIMSP (AIMSP-C) among adolescent basketball players through examining its structural validity using confirmatory factor analysis, evaluating its reliability and convergent validity, and assessing its cultural appropriateness within the Chinese sporting context. A successfully validated AIMSP-C would provide researchers and practitioners with a culturally appropriate tool for understanding and supporting Chinese athletes’ psychological development, particularly during critical developmental periods. Furthermore, this validation would contribute to both theoretical understanding of how cultural factors influence athletic identity formation and practical applications in athlete development programs within China#39;s systematic sports training system.

## Materials & methods

### Participants

A cross-sectional study design was employed. Validation study was conducted to examine the psychometric properties of the Chinese version of the Athletic Identity Measurement Scale Plus (AIMSP-C). Participants were recruited through random sampling from secondary school basketball programs across Shandong Province, China. The sampling strategy ensured representation from diverse competitive levels and training backgrounds.

Eligibility criteria for participation included: (1) current enrollment in secondary school basketball programs; (2) active involvement in organized basketball training and competition; (3) proficiency in reading and comprehending Mandarin Chinese; and (4) voluntary participation with informed consent. Students were excluded if they reported major health conditions interfering with regular basketball participation within the previous six months or lacked competitive experience at minimum city-level competitions.

Sample Size Determination Sample size requirements for confirmatory factor analysis were calculated using Arifin (40) web-based calculator, incorporating parameters established by contemporary structural equation modeling research. The calculation considered the following specifications: anticipated CFI of 0.95, five-factor structure with 4–5 items per factor, expected factor loadings of 0.6, inter-factor correlations of 0.25, significance level (α) of 0.05 (two-tailed), and desired power (1 – β) of 90%. Accounting for potential attrition (15%), the minimum required sample size was determined to be 478 participants. The achieved sample size (N = 604) substantially exceeded this threshold, ensuring robust parameter estimation and stable factor solutions.

### Measures

#### Athletic Identity Measurement Scale Plus (AIMSP).

The AIMSP measures individual differences in athletic identity across multiple dimensions [5]. The AIMSP questionnaire used in this study consisted of 22 items assessing five components of athletic identity: social identity, exclusivity, self-identity, positive affectivity, and negative affectivity. The version used in this study is the original 22-item AIMSP developed by Cieslak II (5), which represents an advancement over the earlier 7-item AIMS [3] and its 10-item revised version by incorporating affective dimensions alongside cognitive components of athletic identity. Each item is scored on an 11-point Likert scale ranging from 1 (strongly disagree) to 11 (strongly agree). The AIMSP has shown good reliability with internal consistency measured by Cronbach#39;s alpha ranging from 0.70 to 0.89 for its subscales [5]. The validity of the AIMSP was examined using CFA and reported marginal goodness of fit (GFI = 0.73, TLI = 0.76, NFI = 0.75) in the original validation study. The AIMSP was developed based on identity theory principles, with items selected through theoretical review and empirical testing. The five-factor structure was established through theoretical review and empirical testing, with items designed to capture both cognitive and emotional aspects of how athletes construct and maintain their athletic role.

### Ethics and procedures

The study received ethical approval from the Universiti Sains Malaysia Human Research Ethics Committee (USM/JEPeM/22050298). The adaptation of the AIMSP into Chinese (AIMSP-C) followed a rigorous translation and validation process adhering to established cross-cultural adaptation guidelines.

It is important to clarify that the AIMSP-C was translated directly from the original English version of the AIMSP as proposed by Cieslak II (5), rather than from any existing Chinese adaptation of related athletic identity scales. This decision was made for three specific reasons: (1) to ensure fidelity to the original five-factor conceptual framework as intended by the scale developer; (2) to maintain the integrity of the complete 22-item structure, including both affective dimensions (Positive Affectivity and Negative Affectivity) that are absent in earlier AIMS versions and their Chinese adaptations; and (3) to avoid potential error accumulation that could arise from translating an already adapted version. It should be noted that the AIMS validated by Zhang and Shao (39) in mainland China assessed a narrower construct using a three-factor model without affective components, whereas the present study employs the full five-dimensional AIMSP framework. This distinction is important for interpreting both the construct validity and the cross-cultural comparability of our findings.

The translation process began with two independent forward translations by bilingual researchers proficient in both English and Mandarin Chinese. These researchers then collaborated to synthesize their translations into an initial Chinese draft through detailed comparison and discussion. A panel of experts, comprising three sports psychologists, two sports science researchers, and two physical education specialists, evaluated this draft version for content validity and cultural appropriateness. Their feedback led to necessary refinements in terminology and expression.

Following expert review, two different bilingual researchers conducted back-translation of the revised Chinese version into English. This back-translated version was compared with the original AIMSP to ensure conceptual and semantic equivalence. Discrepancies were resolved through iterative discussion and modification until achieving satisfactory concordance with the original scale. To enhance face validity, the refined translation underwent cognitive debriefing with a group of twelve native Chinese speakers, who provided feedback on clarity, comprehensibility, and cultural relevance. A subsequent pilot study with fifteen adolescent basketball players confirmed the scale#39;s readability and practical utility.

Data collection occurred between February 2023 and July 2023. The research team distributed informed consent forms to potential participants and their parents or legal guardians, explaining the study#39;s purpose, voluntary nature, and confidentiality measures. Upon obtaining written consent, participants completed the AIMSP-C questionnaire through the Sojump platform, accessing it via personal or parental electronic devices. Prior to administration, researchers provided standardized instructions emphasizing honest responses and the absence of right or wrong answers. The entire data collection process adhered to ethical guidelines for research involving adolescent participants.

Test-retest reliability was assessed using a randomly selected subset of 55 participants from the original sample, who completed the AIMSP-C twice with a 2-week interval between administrations. This sample size was determined using Arifin [40] calculator based on expected ICC of 0.80, minimum acceptable ICC of 0.60, with 80% power and 10% dropout adjustment.

### Statistical analysis

Initial data screening examined univariate and multivariate normality using SPSS 28.0 (IBM Corp, Armonk, NY, USA). The Kolmogorov-Smirnov and Shapiro-Wilk tests assessed univariate normality [41], while Mardia#39;s tests evaluated multivariate normality through skewness and kurtosis coefficients. Visual inspection of histograms and Chi-square versus Mahalanobis distance plots complemented these analyses following recommendations by Tabachnick, Fidell [42].

Confirmatory Factor Analysis (CFA) was conducted using Mplus 8.7 with Maximum Likelihood Robust (MLR) estimator, selected due to the non-normal distribution of the data as recommended by Muthén and Muthén [43]. Model fit was evaluated using multiple indices with criteria established by Hu and Bentler [44]: Comparative Fit Index (CFI > 0.95), Tucker-Lewis Index (TLI > 0.95), Root Mean Square Error of Approximation (RMSEA < 0.06, with 90% confidence intervals), values below 0.10 considered acceptable following Browne and Cudeck [45], and Standardized Root Mean Square Residual (SRMR < 0.08). Factor loadings exceeding 0.40 were considered acceptable following Hair, Sarstedt [46].

Model modifications were implemented using a conservative stepwise approach. Residual correlations were added sequentially based on modification indices, with the modification process stopped when acceptable fit criteria (CFI ≥ 0.95, TLI ≥ 0.95) were achieved to maintain model parsimony and avoid overfitting.

Modification indices (MI) were examined to guide model improvements, with values exceeding 10.0 considered for potential model modifications, as recommended by Byrne [47]. Only theoretically justifiable modifications were implemented, prioritizing residual correlations between items within the same factor or conceptually related items across factors. The largest MI values were addressed first in the sequential modification process, with each modification evaluated for both statistical improvement and theoretical coherence before implementation.

Construct validity assessment included both convergent and discriminant validity. Convergent validity was established through factor loadings (> 0.50), Average Variance Extracted (AVE > 0.50), and Composite Reliability (CR > 0.70) as recommended by Hair, Sarstedt [46]. Discriminant validity was evaluated using the Fornell-Larcker criterion [48], requiring the square root of AVE for each construct to exceed its correlations with other constructs.

Reliability analysis encompassed internal consistency and temporal stability measures. Internal consistency was assessed through CR, with values above 0.70 indicating good reliability [49]. Test-retest reliability was evaluated using two-way mixed effects Intraclass Correlation Coefficients (ICC) as recommended by Koo and Li [50], with a 2-week interval between test and retest administrations, with values interpreted as: poor (< 0.50), moderate (0.50–0.75), good (0.75–0.90), and excellent (> 0.90).

## Results

### Descriptive statistics

A total of 604 secondary school students participated in the study, with an almost equal gender distribution of 301 males (49.8%) and 303 females (50.2%). Participants ranged in age from 12 to 19 years, with a mean age of 15.53 years (SD = 1.42). Most participants were in the senior grades: Junior 1 (5.6%), Junior 2 (4.3%), Junior 3 (6.5%), Senior 1 (28%), Senior 2 (26%), and Senior 3 (29.6%). Regarding sport levels, 28.3% had no specific sport level, 30.5% were at the junior grade level, 23.2% at level 3, 11.1% at level 2, 5% at level 1, and 2% were masters. Participants’ training years varied, with 11.9% having less than one year of training, 34.6% with one year, 31.1% with two years, 14.6% with three years, and 7.8% with more than three years, as detailed in Table 1.

**Table 1:** Demographic Information and Frequency of Participants.

Category	Frequencies	Percentage	Mean (SD)
**Gender**			
Male	301	49.8	
Female	303	50.2	
**Grade**			
Junior 1	34	5.6	
Junior 2	26	4.3	
Junior 3	39	6.5	
Senior 1	169	28	
Senior 2	157	26	
Senior 3	179	29.6	
**Age**			15.53(1.42)
**Training Years**			
Less Than One Year	72	11.9	
One Year	209	34.6	
Two Years	188	31.1	
Three Years	88	14.6	
More Than Three Years	47	7.8	
**Sport Level**			
None	171	28.3	
Junior grade	184	30.5	
Level 3	140	23.2	
Level 2	67	11.1	
Level 1	30	5	
Master	12	2	

Table 2 shows the distribution of the items’ scores for the Chinese version of the Athletic Identity Measurement Scale - Plus (AIMSP). The mean (SD) scores for all items ranged between 4.58 (1.81) for AIMSP15 and 6.92 (3.06) for AIMSP6.In the 11 options, 1 = Strongly Disagree, 6 = Neutral, and 11 = Strongly Agree. Most participants provided their responses in the higher categories of the scale. Specifically, the highest percentages of participants answered “1” for AIMSP19 (9.6%), “2” for AIMSP21 (14.6%), “3” for AIMSP15 (12.3%), “4” for AIMSP19 (26.5%), “5” for AIMSP15 (24.7%), “6” for AIMSP10 and AIMSP16 (both 24.7%), “7” for AIMSP4 (30.3%), “8” for AIMSP5 (33.1%), “9” for AIMSP7 (14.1%), “10” for AIMSP6 (16.4%), and “11” for AIMSP7 (12.9%).

**Table 2:** Distribution of the items’ score for Chinese version of AIMSP scale.

Items	Mean (SD)	Range	Skewness	Kurtosis	% Agree*	% Neutral**	% Disagree***
AIMSP1	5.13(2.14)	1-11	0.15	−0.82	45	23.2	31.8
AIMSP2	5.05(2.07)	1-11	0.31	−0.65	48.5	20.4	31.1
AIMSP3	5.18(2.13)	1-11	0.22	−0.58	35.7	21.5	42.8
AIMSP4	6.04(2.57)	1-11	−0.28	−0.95	57.8	5.5	36.7
AIMSP5	6.19(2.72)	1-11	−0.31	−1.02	57.9	3.1	39
AIMSP6	6.92(3.06)	1-11	−0.48	−1.15	60.9	8.4	30.7
AIMSP7	6.72(3.19)	1-11	−0.45	−1.22	58.1	8.4	33.5
AIMSP8	6.11(2.52)	1-11	−0.22	−0.89	58.1	6.3	35.6
AIMSP9	6.75(3.12)	1-11	−0.41	−1.18	58.1	8.9	33
AIMSP10	5.31(2.18)	1-11	0.35	−0.48	45.9	24.7	29.4
AIMSP11	5.29(2.04)	1-11	0.18	−0.52	47.2	12.7	40.1
AIMSP12	6.71(3.11)	1-11	−0.42	−1.16	57	9.9	33.1
AIMSP13	5.08(2.06)	1-11	0.32	−0.51	46	19.9	34.1
AIMSP14	5.10(2.19)	1-11	0.29	−0.65	34.6	21.5	43.9
AIMSP15	4.58(1.81)	1-11	0.58	−0.12	27.3	24.7	48
AIMSP16	5.28(2.20)	1-11	0.31	−0.68	45	24.7	30.3
AIMSP17	6.21(2.68)	1-11	−0.27	−0.98	56.3	7.8	35.9
AIMSP18	6.76(3.15)	1-11	−0.44	−1.2	59.1	7.5	33.4
AIMSP19	4.87(2.17)	1-11	0.45	−0.51	35.4	13.1	51.5
AIMSP20	6.17(2.67)	1-11	−0.25	−0.96	56.8	3.8	39.4
AIMSP21	5.17(1.94)	1-11	0.15	−0.41	44.4	18.7	36.9
AIMSP22	5.28(2.23)	1-11	0.31	−0.68	45.4	19.9	34.7

Note: *% Agree = responses 7–11 (above neutral); **% Neutral = response 6 (neutral point); ***% Disagree = responses 1–5 (below neutral); Scale: 1 = Strongly Disagree, 6 = Neutral, 11 = Strongly Agree.

Table 2 presents the descriptive statistics for all AIMSP-C items. Most items showed approximately normal distribution, with skewness values ranging from −0.48 to 0.58 and kurtosis values from −1.22 to −0.12. Response patterns indicated that participants generally used the full range of the 11-point scale, with mean scores ranging from 4.58 (AIMSP15) to 6.92 (AIMSP6).

### Confirmatory factor analysis

**Fig 1:**
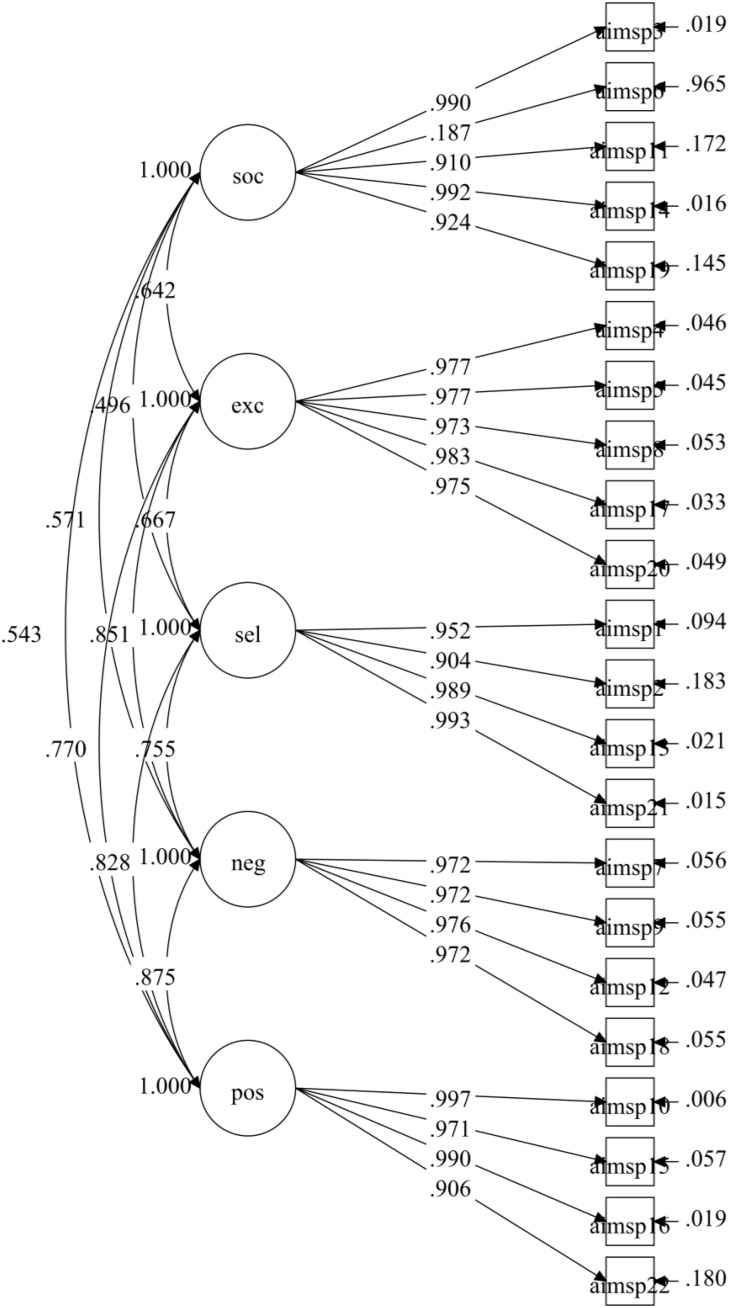
CFA Diagram of the AIMSP-C Initial model. Note: soc = Social Identity, exc = Exclusivity, sel = Self-Identity, neg = Negative Affectivity, pos = Positive Affectivity..

Confirmatory Factor Analysis revealed that the initial model of AIMSP-C demonstrated suboptimal fit indices (CFI = 0.929, TLI = 0.918, SRMR = 0.064, RMSEA = 0.131 [90% CI: 0.126, 0.136]), as illustrated in Fig 1. Model modifications were implemented using a conservative stepwise approach based on modification indices. Item AIMSP6 was removed due to poor factor loading (0.187), which fell well below the acceptable threshold of 0.40 recommended by Hair, Sarstedt [46]. Additionally, examination of the item content “I need to participate in sport to feel good about myself” revealed potential cross-loading issues with other dimensions and cultural adaptation concerns in the Chinese context. The item#39;s removal was further supported by modification indices suggesting poor model fit contribution and its minimal impact on the Social Identity factor#39;s overall reliability. Subsequently, residual correlations were added sequentially until acceptable fit indices were achieved. The final model included two residual correlations between theoretically related items (AIMSP14-AIMSP3, AIMSP20-AIMSP5), stopping when CFI and TLI values reached approximately 0.95 to maintain model parsimony and avoid overfitting.

**Fig 2:**
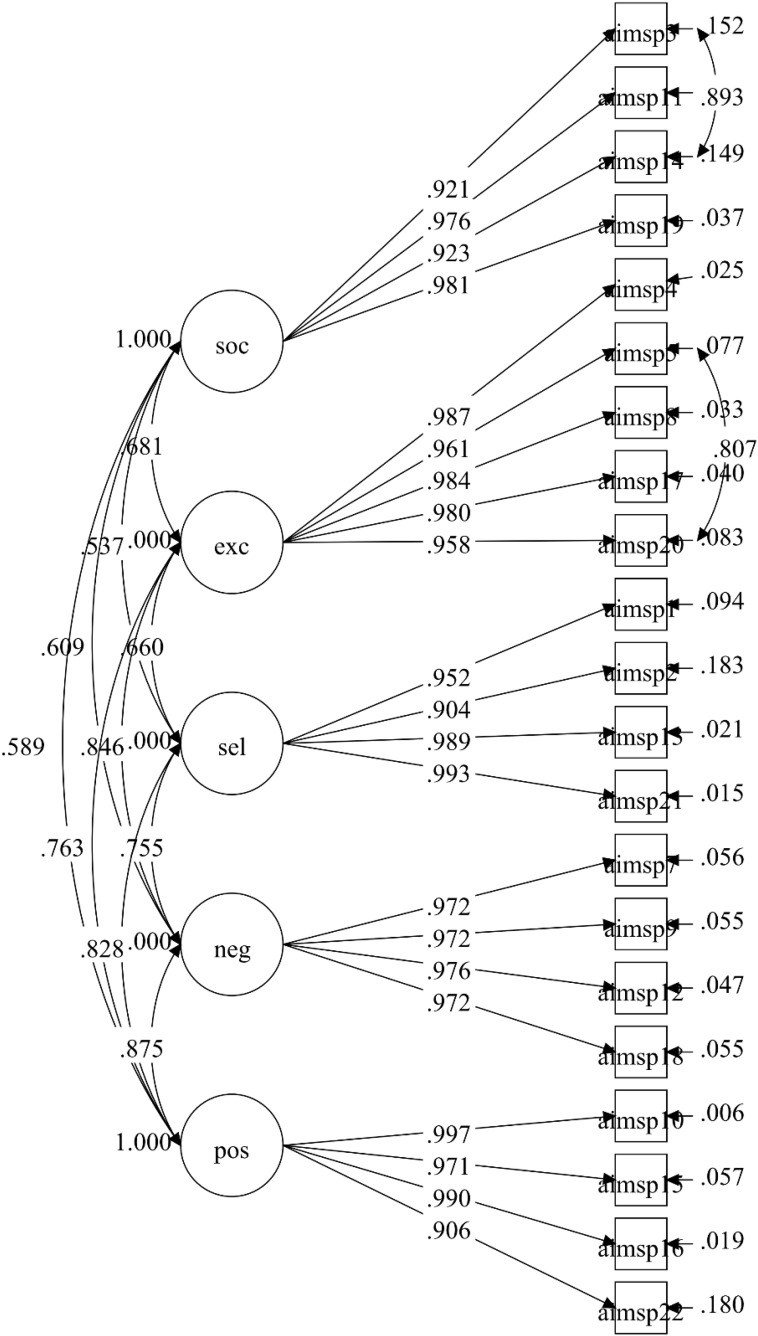
CFA Diagram of the AIMSP-C Final model. Note: soc = Social Identity, exc = Exclusivity, sel = Self-Identity, neg = Negative Affectivity, pos = Positive Affectivity..

The modified model demonstrated acceptable fit indices (CFI = 0.959, TLI = 0.952, SRMR = 0.083, RMSEA = 0.091 [90% CI: 0.086, 0.096]), meeting established criteria for acceptable model fit while maintaining model parsimony (Table 3). The CFI, TLI, and SRMR met their respective thresholds, while the RMSEA value of 0.091 fell within the acceptable range below 0.10 [45], supporting the structural validity of the final AIMSP-C model (Table 4, Fig 2).

**Table 3:** CFA Fit Indices for the AIMSP-C (Initial and Final models).

Model	CFI	TLI	SRMR	RMSEA (90%CI)
Initial model^a^	0.929	0.918	0.064	0.131 (0.126, 0.136)
Final model^b^	0.959	0.952	0.083	0.091 (0.086, 0.096)

Note: Modelb Deleted: AIMSP6.

Model with correlated items’ residual: AIMSP14 WITH AIMSP3; AIMSP20 WITH AIMSP5.

**Table 4:** Factor Loadings of the AIMSP-C for Initial and Final Models.

Factors/items	Factor loading	
	Initial model	Final model
SOC		
AIMSP3	0.990	0.921
AIMSP6	0.187	Deleted
AIMSP11	0.910	0.976
AIMSP14	0.992	0.923
AIMSP19	0.924	0.981
EXC		
AIMSP4	0.977	0.987
AIMSP5	0.977	0.961
AIMSP8	0.973	0.984
AIMSP17	0.983	0.980
AIMSP20	0.975	0.958
SEL		
AIMSP1	0.952	0.952
AIMSP2	0.904	0.904
AIMSP13	0.989	0.989
AIMSP21	0.993	0.993
NEG		
AIMSP7	0.972	0.972
AIMSP9	0.972	0.972
AIMSP12	0.976	0.976
AIMSP18	0.972	0.972
POS		
AIMSP10	0.997	0.997
AIMSP15	0.971	0.971
AIMSP16	0.990	0.990
AIMSP22	0.906	0.906

Note: SOC = Social Identity, EXC = Exclusivity, SEL = Self-Identity, NEG = Negative Affectivity, POS = Positive Affectivity.

### Convergent and discriminant validity

The AIMSP-C demonstrated strong psychometric properties across all dimensions. Factor loadings in the final model remained robust, ranging from 0.906 to 0.997 across all items, indicating strong item-factor relationships. The Social Identity dimension showed high loadings (0.921–0.981), as did Exclusivity (0.958–0.987), Self-Identity (0.904–0.993), Negative Affectivity (0.972–0.976), and Positive Affectivity (0.906–0.997). The minor variations between initial and final models’ factor loadings suggest stability in the measurement structure.

Construct reliability analysis revealed exceptional internal consistency, with composite reliability (CR) values ranging from 0.974 to 0.989 across all factors. Internal consistency was further evaluated using Cronbach#39;s alpha and McDonald#39;s omega coefficients. For the final model, Cronbach#39;s alpha values were: Social Identity (α = 0.974), Exclusivity (α = 0.989), Self-Identity (α = 0.979), Negative Affectivity (α = 0.986), and Positive Affectivity (α = 0.982). McDonald#39;s omega coefficients showed nearly identical patterns: Social Identity (ω = 0.974), Exclusivity (ω = 0.989), Self-Identity (ω = 0.979), Negative Affectivity (ω = 0.986), and Positive Affectivity (ω = 0.983). Convergent validity was established through Average Variance Extracted (AVE) values consistently exceeding 0.50 (range: 0.904–0.949), indicating that the items effectively represent their respective constructs (Table 5). All reliability coefficients exceeded recommended thresholds, with values greater than 0.97 across all dimensions, demonstrating excellent internal consistency (Table 6).

**Table 5:** Correlation Matrix and Discriminant Validity of the AIMSP-C (Initial and Final models).

Initial	CR.	AVE.	SOC	EXC	SEL	NEG	POS
SOC	0.924	0.736	0.858				
EXC	0.991	0.955	0.642*	0.977			
SEL	0.979	0.922	0.496*	0.667*	0.960		
NEG	0.986	0.947	0.571*	0.851*	0.755*	0.973	
POS	0.983	0.934	0.543*	0.770*	0.828*	0.875*	0.967
Final							
SOC	0.974	0.904	0.951				
EXC	0.989	0.949	0.681*	0.974			
SEL	0.979	0.922	0.537*	0.66*	0.960		
NEG	0.986	0.947	0.609*	0.846*	0.755*	0.973	
POS	0.983	0.935	0.589*	0.763*	0.828*	0.875*	0.967

Note: * p < 0.05, CR = Composite Reliability, AVE = Average Variance Extracted, SOC = Social Identity, EXC = Exclusivity, SEL = Self-Identity, NEG = Negative Affectivity, POS = Positive Affectivity.

**Table 6:** Internal Consistency Reliability Coefficients for AIMSP-C Final Model.

Dimension	Cronbach’s α	McDonald’s ω	CR
Social Identity	0.974	0.974	0.974
Exclusivity	0.989	0.989	0.989
Self-Identity	0.979	0.979	0.979
Negative Affectivity	0.986	0.986	0.986
Positive Affectivity	0.982	0.983	0.983

### Test-retest reliability

The test-retest reliability of the AIMSP-C was evaluated using the Intraclass Correlation Coefficient (ICC) values for the five subscales, indicating excellent stability over time. The ICC values were as follows: Social Identity (0.936), Exclusivity (0.989), Self-Identity (0.985), Negative Affectivity (0.988), and Positive Affectivity (0.973). These values demonstrate strong reliability, with ICC values greater than 0.70 indicating good reliability and values greater than 0.90 indicating excellent reliability (Table 7).

**Table 7:** Intraclass Correlation Coefficients (ICC) for the AIMSP-C.

Dimension	ICC
Social Identity	0.936
Exclusivity	0.989
Self-Identity	0.985
Negative Affectivity	0.988
Positive Affectivity	0.973

## Discussion

The present study aimed to validate the Chinese version of the Athletic Identity Measurement Scale Plus (AIMSP-C) among adolescent athletes in mainland China. Confirmatory factor analysis conducted on a sample of 604 secondary school students supported the five-factor structure of AIMSP, which aligns with Brewer, Van Raalte (3) original conceptualization of athletic identity as a multidimensional construct. After minor modifications, including the deletion of one item (AIMSP6) and allowing several item residuals to correlate, the final model demonstrated acceptable fit indices (CFI = 0.959, TLI = 0.952, RMSEA = 0.091, SRMR = 0.083).

The model modification process followed conservative principles, with residual correlations added only until acceptable fit criteria were met (CFI and TLI ≥ 0.95), rather than pursuing optimal fit indices. This approach prioritized model parsimony and generalizability over marginal fit improvements, addressing potential concerns about overfitting that can compromise model replicability across different samples. The final model included only two residual correlations, demonstrating a more parsimonious solution compared to extensive post-hoc modifications.

### Theoretical interpretation of residual correlations

The two residual correlations included in the final model (AIMSP14-AIMSP3, AIMSP20-AIMSP5) can be theoretically justified within identity theory frameworks. The correlation between AIMSP14 (“Others see me mainly as an athlete”) and AIMSP3 (“Most of my friends are athletes”) reflects the interconnected nature of social identity formation, where external recognition and peer networks mutually reinforce each other. Similarly, the correlation between AIMSP20 (“I spend more time thinking about sport than anything else”) and AIMSP5 (“Sport is the most important part of my life”) represents shared variance in exclusivity-related cognitions, reflecting the cognitive overlap in how athletes prioritize sport in their identity hierarchy.

### Psychometric properties and cross-cultural validation

The psychometric properties of AIMSP-C demonstrated robust characteristics across cultures. Compared to the Iranian validation study [36], which yielded a three-factor structure with internal consistency of 0.88 among 384 physical education majors, our study achieved superior reliability indices (CR: 0.974–0.989) and validity indicators (AVE: 0.904–0.947). Similarly, while [37] Hong Kong validation maintained the original factor structure, our mainland China sample showed stronger psychometric properties, suggesting potential regional variations in athletic identity manifestation within Chinese cultural contexts. The Pakistani validation [38] also supported a five-factor structure but with relatively lower fit indices (χ²/df = 2.69, CFI = 0.96, TLI = 0.98), possibly due to their smaller sample size.

### Cultural manifestations of athletic identity

A distinctive finding emerged in the relationship patterns among the five dimensions. Social identity showed strong correlations with both self-identity and exclusivity, supporting [39] findings regarding the interconnected nature of identity components in Chinese athletes. This pattern reflects the collective orientation in Chinese sports culture, where individual athletic identity development is closely tied to social recognition and team integration. The exclusivity dimension demonstrated particularly interesting characteristics in our sample. While maintaining strong psychometric properties, its relationship with other dimensions suggests a uniquely Chinese manifestation of athletic role commitment. This finding extends our understanding of how cultural context influences the balance between athletic and other life roles, a consideration particularly relevant in China#39;s sports-education integration system.

The affective components (both positive and negative) showed higher inter-correlations than typically reported in Western samples, suggesting a more integrated emotional experience among Chinese athletes. This finding aligns with research on cultural differences in emotional processing and expression, particularly in athletic contexts where performance pressure and achievement expectations are culturally mediated. Of particular significance was the exceptional test-retest reliability demonstrated by AIMSP-C, with ICC values ranging from 0.936 to 0.989 across dimensions. This temporal stability is crucial for longitudinal tracking of athletic identity development, especially during the critical adolescent period when identity formation intersects with intensive athletic training.

### Practical applications and limitations

From a practical standpoint, the validated AIMSP-C provides several important applications. It offers coaches and sport psychologists a reliable instrument for evaluating athletic identity development in Chinese youth athletes, enables targeted interventions addressing specific aspects of athletic identity, and demonstrates the scale#39;s sensitivity to Chinese cultural nuances while maintaining measurement rigor. While this study establishes strong support for AIMSP-C#39;s utility, certain limitations warrant consideration. The current validation focused primarily on internal structure and reliability; future research should examine convergent and divergent validity through correlations with established measures of related constructs (e.g., self-esteem, sport commitment). Additionally, criterion validity should be assessed by examining relationships with objective performance measures and behavioral indicators of athletic identity. The sample composition, particularly the high proportion of participants without specific competitive levels (28.3%), suggests the need for additional validation with elite athletes. Furthermore, the geographic concentration in one region may limit generalizability across China#39;s diverse sporting contexts.

### Future directions

Future research should include longitudinal studies tracking athletic identity development across career stages, cross-validation studies with elite athletes, investigation of regional variations within China, and examination of sport-specific identity patterns. In conclusion, the successful validation of AIMSP-C provides a robust tool for understanding athletic identity in Chinese youth athletes while contributing to the broader theoretical framework of athletic identity measurement across cultures. The findings support the universal aspects of athletic identity while highlighting culturally specific manifestations, particularly in the Chinese sporting context where athletic development is uniquely integrated with academic education.

## Conclusion

This study validates the AIMSP-C among Chinese adolescent basketball players, addressing the critical need for culturally appropriate athletic identity measurement tools. The findings confirm the five-factor structure of athletic identity while revealing distinctive characteristics within the Chinese sporting context. The AIMSP-C demonstrated acceptable psychometric properties, with robust reliability, validity, and test-retest stability, meeting established criteria for cross-cultural validation.

The strong correlations between social identity and other dimensions reflect the collective orientation in Chinese sports culture, highlighting how cultural context shapes athletic identity manifestation. This validated instrument provides researchers and practitioners with a reliable tool for understanding and supporting Chinese athletes’ psychological development, particularly within the sports-education integration system. Longitudinal validation studies will be particularly important to establish the scale#39;s temporal stability and sensitivity to developmental changes in athletic identity formation. Future research should extend validation to elite athletes and examine regional variations within China#39;s sporting landscape.

## Supporting information

S1 File Chinese version of the Athletic Identity Measurement Scale Plus (AIMSP-C). The complete 22-item questionnaire in Mandarin Chinese with English translation.
(PDF)

S2 File Dataset. The raw data underlying the findings described in this manuscript.
(XLSX)
