# Supplementary material for: Psychometric properties of the Chinese version of the Athletic Identity Measurement Scale Plus: A confirmatory study on Chinese adolescents
Source: PLoS One. 2026 Apr 13;21(4):e0345181. doi: 10.1371/journal.pone.0345181 (PMC13075664; doi:10.1371/journal.pone.0345181)
Supplement: S1 File — The complete 22-item questionnaire in Mandarin Chinese with English translation. (PDF) [file pone.0345181.s001.pdf]

Athlete Identity Measurement Scale-Plus (AIMS-Plus)

Please **circle** the number that best reflects the extent to which you agree or disagree with each statement in relation to your own sports participation.

Rate each item using the following scale:

Strongly disagree	Neutral	Strongly agree
0 1 2 3 4	5 6 7 8 9	10

1	I consider myself an athlete.
2	I have many goals related to sport.
3	Most of my friends participate in sport
4	Sport is the most important part of my life.
5	I spend more time thinking about sport than anything else.
6	Other people see me as an athlete.
7	I feel bad about myself when I play poorly in practice or competition.
8	Sport is the only important thing in my life.
9	I would be very depressed if I were injured and could not compete in sport.
10	When I am participating in sport, I am happy.
11	My family expects me to participate in sport.
12	1 feel badly when I fail to meet my athletic goals.
13	Being an athlete is who I am and I want to make a career of sport.
14	It is important that other people know about my sport involvement.
15	I get a sense of satisfaction when participating in sport.
16	My participation in sport is a very positive part of my life.
17	I typically organize my day so I can participate in sports.
18	I would be very depressed if I were cut from the team and could not compete in sport.
19	I participate in sport for the recognition/fame.
20	My sports involvement has influenced my day-to-day decision-making.
21	Being an athlete is an important part of who I am.
22	I feel good about myself when I play well in practice or competition.

中文版：运动员身份测量量表-Plus

请选出最能反映您对与您自己的运动参与有关的各项陈述的同意或不同意程度的数字。

	强烈不同意	1	2	3	4	中立	6	7	8	9	强 烈同意
1.我认为自己是一名运动员。	○	○	○	○	○	○	○	○	○	○	○
2.我有许多与体育运动有关的目标。	○	○	○	○	○	○	○	○	○	○	○
3.我的大多数朋友都参加了体育运动。	○	○	○	○	○	○	○	○	○	○	○
4.运动是我生命中最重要的部分。	○	○	○	○	○	○	○	○	○	○	○
5.我花更多的时间思考体育运动的问题，而不是其他问题。	○	○	○	○	○	○	○	○	○	○	○
6.其他人认为我是一名运动员。	○	○	○	○	○	○	○	○	○	○	○
7.当我在训练或比赛中发挥不佳时，我对自己感到很难过。	○	○	○	○	○	○	○	○	○	○	○
8.体育运动是我生活中唯一重要的事情。	○	○	○	○	○	○	○	○	○	○	○
9.如果我受伤了不能参加体育比赛，我会非常沮丧。	○	○	○	○	○	○	○	○	○	○	○
10.当我参加体育活动时，我很高兴。	○	○	○	○	○	○	○	○	○	○	○
11.我的家人希望我参加体育运动。	○	○	○	○	○	○	○	○	○	○	○
12.当我没有达到我的运动目标时，我感到很难过。	○	○	○	○	○	○	○	○	○	○	○
13.我是一名运动员，我想以体育为职业	○	○	○	○	○	○	○	○	○	○	○
14.让其他人知道我在参与体育运动是很重要的。	○	○	○	○	○	○	○	○	○	○	○
15.我在参加体育活动时有一种满足	○	○	○	○	○	○	○	○	○	○	○

感											
16.我参与体育活动是我生活中非常积极的一部分。	○	○	○	○	○	○	○	○	○	○	○
17.我通常会安排我的一天，以便我能够参加体育活动。	○	○	○	○	○	○	○	○	○	○	○
18.如果我被开除出球队，不能参加比赛，我会非常沮丧。	○	○	○	○	○	○	○	○	○	○	○
19.我参加体育活动是为了获得认可/名声。	○	○	○	○	○	○	○	○	○	○	○
20.我参与体育运动影响了我对日常事务的决策。	○	○	○	○	○	○	○	○	○	○	○
21.成为一名运动员是我的人生中一个重要的一部分。	○	○	○	○	○	○	○	○	○	○	○
22.当我在练习或比赛中发挥出色时，我对自己感觉良好。	○	○	○	○	○	○	○	○	○	○	○
